# Facteurs associés au premier recours thérapeutique des mères d’enfants de 0-5 ans au Bénin

**DOI:** 10.11604/pamj.2025.52.38.45604

**Published:** 2025-09-24

**Authors:** Kougbéssi Gélase Atiogbe, Nicolas Gaffan, Annonciat Sèmèvo Aviansou, Ghislain Emmanuel Sopoh, Edgard-Marius Ouendo

**Affiliations:** 1 Institut Régional de Santé Publique de Ouidah, Université d'Abomey-Calavi, Ouidah, Bénin,; 2 Ministère de la Santé, Cotonou, Bénin

**Keywords:** Enfants 0-5 ans, recours-thérapeutique, relais-communautaire, intervention-à-haut-impact, Bénin, Children under 5 years, therapeutic recourse, community health worker, high-impact intervention, Benin

## Abstract

**Introduction:**

l'objectif de cette étude était de déterminer les facteurs associés au premier recours thérapeutique des mères d'enfants de moins de cinq ans dans les zones de mise en œuvre des interventions traceurs du paquet d'intervention à haut impact communautaire au Bénin.

**Méthodes:**

étude transversale analytique portant sur les mères et leur plus jeune enfant de moins de cinq ans. Un échantillonnage aléatoire à plusieurs degrés a été utilisé. La variable dépendante était le premier recours thérapeutique de la mère lors du dernier épisode morbide de son plus jeune enfant. Une régression logistique multivariée a été réalisée afin d'identifier les facteurs associés au premier recours thérapeutique utilisé par les mères.

**Résultats:**

quatre cent quarante-huit mères d'enfants de moins de cinq ans ont été incluses dans l'étude. Suite au dernier épisode morbide de leur plus jeune enfant, 52,45% des mères ont eu premièrement recours à la médecine moderne ou aux relais communautaires contre 47,55% qui ont pratiqué une automédication, recouru aux praticiens de la médecine traditionnelle ou n'ont pas recherché les soins. Les facteurs associés au recours aux soins des mères d'enfants de moins de cinq ans en cas d'épisode morbide de leur plus jeune enfant étaient la confession chrétienne ou musulmane, connaissance d'un relais communautaire par la mère, disponibilité de toilette dans leur domicile et résidence dans les départements du Nord du Bénin.

**Conclusion:**

les facteurs identifiés soulignent l'importance d'intervenir sur des éléments modifiables tels que la promotion de l'action des relais communautaires pour améliorer le recours à leurs prestations.

## Abstract (en)

**Introduction:**

the purpose of this study was to identify the factors associated with mothers’ first therapeutic recourse for children under five years in areas implementing tracer interventions of the high-impact community package in Benin.

**Methods:**

we conducted an analytical cross-sectional study involving mothers and their youngest child under five years of age. A multistage random sampling method was applied. The dependent variable was mother’s first therapeutic recourse during her youngest child’s most recent illness episode. A multivariate logistic regression was performed to identify factors associated with mothers’ first therapeutic recourse.

**Results:**

a total of 448 mothers of children under five years were included. Following the youngest child’s most recent illness episode, 52.45% of mothers initially resorted to modern medicine or community health workers, while 47.55% engaged in self-medication, consulted traditional medicine practitioners, or did not seek care at all. Factors associated with mothers’ health care’ seeking for children under five during the most recent illness episode of their youngest child included Christian or Muslim faith, knowledge of a community health worker, availability of a household toilet, and residence in northern regions of Benin.

**Conclusion:**

the identified factors highlight the importance of addressing modifiable elements such as promoting the role of community health workers to improve mothers’ timely use of their services.

## Introduction

La mission principale du système de santé reste l'amélioration du bien-être socio-sanitaire des communautés. A cet effet, le succès de la couverture sanitaire universelle dépend de l'accès de tous à des soins sûrs, efficaces, fondés sur des données probantes et axés sur la personne [1]. Comme le souligne les objectifs de développement durables (ODD), l'accès et le recours aux soins sont des enjeux majeurs de santé publique, mais sont également complexes, car dépendent de facteurs multiples [2,3]. En 2019, on estime que 5,2 millions d'enfants âgés de moins de cinq ans sont décédés, le plus souvent de maladies qui pourraient être évitées ou traitées [4]. Pour remédier à cette situation, les pays ont entrepris ces dernières années de nombreuses réformes dans le domaine de la santé, en mettant en place diverses stratégies avec des actions au niveau de la communauté. Mais force est de constater que l'accès et le recours aux soins demeurent toujours insuffisants dans plusieurs régions du monde [5].

En Afrique, les gouvernements sont conscients de cette situation et désireux d'y remédier, développant au cours des dernières années des stratégies volontaristes pour accélérer l'amélioration de l'état de santé des populations [6]. C'est la mise en œuvre du PIHI Com gouvernement du Bénin a adopté l'approche dénommée Paquet d'Interventions à Haut Impact au niveau Communautaire (PIHI Com) qui est un ensemble d'interventions sanitaires fondées sur des données factuelles. En 2009 et 2010, à l'issue de plusieurs ateliers, un consensus a été fait sur les interventions à mener ainsi que les indicateurs de suivi du PIHI Com et les directives nationales de mise en œuvre élaborés, pour faciliter son opérationnalisation [7]. La mise en œuvre du PIHI Com a démarré par une phase pilote menée dans seize (16) zones sanitaires sur les trente-quatre (34) que compte le pays. L'objectif du PIHI Com était de mener des interventions ayant un impact sur la réduction de la mortalité maternelle, néonatale et infanto-juvénile. Ces interventions sont scindées en deux groupes: le Paquet d'Interventions à Haut Impact Complémentaire (PIHI-C) mise en œuvre à petite échelle mais pas dans les formations sanitaires et le Paquet d'Interventions à Haut Impact de Base (PIHI-B) mise en œuvre dans les formations sanitaires jusqu'au niveau communautaire. Parmi ces interventions, six (6) ont été identifiées comme « traceurs » dont la mise en œuvre a permis de détecter et d'analyser les goulots d'étranglement des prestations de services de santé. Ainsi, pour accroître l'offre de services de santé, principalement dans le domaine des soins primaires, la stratégie PIHI entend assurer le libre accès aux services de santé à la communauté en général. Néanmoins, selon l'annuaire statistique de 2022, le taux de fréquentation des services de santé demeure à 61,4% [8]. Pour améliorer l'accès aux prestations et services de soins, il faut trouver des réponses adaptées et adéquates.

Cet article a exploré la mesure selon laquelle les interventions du PIHI ont influencé les pratiques des communautés ciblées en ce qui concerne le recours initial aux soins. L'objectif principal de cette étude était de déterminer les facteurs associés au premier recours thérapeutique des mères d'enfants de moins de cinq ans dans les zones de mise en œuvre des interventions traceurs du paquet d'intervention à haut impact communautaire au Bénin.

## Méthodes

**Cadre d'étude:** le Bénin est un Etat de la région ouest-africaine qui couvre une superficie de 114763 km^2^ et limité au Sud par l'Océan Atlantique, à l'Ouest par le Togo, au Nord par le Burkina-Faso et le Niger et à l'Est par le Nigeria avec une population de 10008749 habitants. Au plan administratif, le pays compte 12 départements [9]: Alibori, Atacora, Atlantique, Borgou, Collines, Couffo, Donga, Littoral, Mono, Ouémé, Plateau et Zou. Ces départements sont divisés en 77 communes, elles-mêmes subdivisées en 546 arrondissements comportant 5295 villages et quartiers de ville. Six communes Banikoara, Bembérèkè, Kalalé, Malanville, Nikki, et Sinendé abritent la phase pilote de la mise en œuvre de la nouvelle politique de la santé communautaire qui est en pleine extension [10].

**Type d'étude:** il s'est agi d'une étude transversale analytique.

**Population d'étude:** la population d'étude était constituée des mères d'enfants de moins de cinq ans résidant dans les zones d'intervention du PIHI Com au Bénin. Les mères d'enfants éligibles qui n'avaient pas donné leur consentement oral à participer à l'étude n'ont pas été incluses. Calcule de la taille de l'échantillon [11]


$$n=\frac{t^{2}\times p\times \left(1-p\right)}{m^{2}}\;\;\text{Ou}\;n=\frac{\varepsilon^{2}\times p\times qe}{i^{2}}$$

n=((1,96)^2^x0,39x0,61)/(0,05)^2^, n= 366; n= taille de l'échantillon qui représente l'effectif des enfants de 0 à 5 ans dont on va administrer le questionnaire aux mères d'enfants; t ou ε= écart réduit du niveau de confiance ou est l'écart réduit pour un risque α égal à 5%= 1,96; p= prévalence de paludisme pour les enfants de 6-59 mois [12] q= 1 - p, m= marge d'erreur acceptable ou i= degré de précision qui est 5%

Nous avons: n= taille de l'échantillon requise t ou ε = niveau de confiance à 95% (écart réduit= 1,96) p= prévalence estimée (39%) soit p= 0,39 q= 1-0,39= 0,61; m= marge d'erreur acceptable 5% (0,05) ou i= degré de précision qui est 5%. La taille de l'échantillon sera majorée de 10% pour les non-réponses, soit n=402 ménages.

**Méthodes et techniques d'échantillonnage:** au niveau de chaque département, un tirage aléatoire simple d'une commune ayant bénéficié des interventions du PIHI Com a été opéré. Ce tirage s'est appuyé sur une cartographie récente des interventions et des partenaires du PIHI Com que nous avons réalisés dans le cadre de ses travaux. Le département du Couffo n'a pas été retenu (car n'ayant pas bénéficié des interventions du PIHI Com). Par suite, au niveau de chaque commune, un arrondissement, puis un village ou quartier de ville ont été successivement sélectionnés de façon aléatoire. Les onze villages ou quartiers de ville ainsi sélectionnés sont Arbonga, Koutatiegou, Zoungbomè, Bonkora, Gbowele, Kodowari, Kowegbo, Doguia, Tanzoun, Okoffin et Hounviguèli. Par la suite, à partir d'un croisement situé au centre du village ou quartier de ville, l'enquêteur a choisi une direction au hasard en jetant un stylo en l'air; la direction à prendre étant indiquée par la pointe du stylo. En suivant cette direction, il a procédé à la sélection des ménages. La première maison a été tirée au hasard parmi les maisons. Au niveau d'un ménage visité, toutes les mères d'enfants de moins de cinq ans étaient éligibles pour être enquêtées. Ensuite, on a procédé de proche en proche jusqu'à atteindre le nombre de cible à enquêter dans chaque village ou quartier de ville. La taille minimale de l'échantillon calculée selon la formule de Schwartz était de 435 mères d'enfants de moins de cinq ans, soit au moins 40 par village [11]. Au total, 448 mères d'enfants de moins de cinq ans ont été incluses dans l'étude.

### Variables d'étude

**Variable dépendante:** elle était le premier recours thérapeutique de la mère lors du dernier épisode morbide de son plus jeune enfant. Elle comprenait les modalités suivantes: « automédication moderne », « automédication traditionnelle », « médecine moderne », « relais communautaire », « médecine traditionnelle » et « pas de recherche de soins ». « L'automédication moderne » regroupait les cas où les mères sans consultation d'un professionnel de santé, ont utilisé des « médicaments modernes » (comprimés, sirops, pommades, etc.) disponibles dans leurs domiciles ou achetés pour l'occasion. « L'automédication traditionnelle » désignait les cas où les mères ont eu à utiliser des « produits traditionnels » disponibles à la maison ou achetés pour traiter l'enfant sans consultation d'un praticien de la médecine traditionnelle. La modalité « médecine moderne » regroupait les cas où l'enfant a été reçu dans une formation sanitaire privée ou publique pour consultation par un professionnel de santé. La modalité « relais communautaire » regroupait les cas où un relais communautaire a été sollicité pour la prise en charge de l'enfant. La modalité « médecine traditionnelle » désignait les cas où la mère a consulté un praticien de la médecine traditionnelle. Enfin, la modalité « Pas de recherche de soins » regroupait les cas où la mère n'a pas eu recours aux possibilités susmentionnées. Par la suite, la variable a été dichotomisée de la manière suivante: recours à la médecine moderne ou au relais communautaire et les autres recours.

**Variables indépendantes:** elles étaient les suivantes: niveau d'instruction de la mère (non-instruite/alphabétisée, primaire, secondaire et plus), situation matrimoniale de la mère (célibataire, mariée), activité génératrice de revenu de la mère (non, oui), religion de la mère (aucune, christianisme, islam, traditionnelle et autres), connaissance de la mère d'un relais communautaire (non, oui), connaissance de la mère du PIHI Com (non, oui), taille du ménage (≤ 5, > 5), MII non-déchirés dans le ménage (non, oui), disponibilité de latrine dans le domicile (non, oui), âge du plus jeune enfant en mois (< 12, 12-23, 24-35, 36-47, 48-59), sexe du plus jeune enfant (féminin, masculin), rang de naissance du plus jeune enfant (1, 2 et 3 et plus), région (sud, centre et nord).

**Collecte et traitement des données:** les données ont été collectées par le biais d'un questionnaire structuré, au cours d'un entretien. Une fois les données collectées, elles ont été saisies avec Epi Info 7 et analysées sous Stata/SE 15.1. Après la saisie des données, un apurement de la base a été réalisé.

**Analyse des données:** dans un premier temps, les différentes variables à l'étude ont été décrites. Les variables quantitatives ont été présentées suivant leurs paramètres de tendance centrale et de dispersion. La moyenne et l'écart-type ont été utilisés lorsque la distribution est normale et lorsque la distribution est asymétrique, la médiane et l'intervalle interquartile ont été utilisés. Quant aux variables qualitatives, elles ont été décrites en calculant les effectifs et pourcentages de leurs modalités. Une régression logistique multivariée a été réalisée afin d'identifier les facteurs associés au premier recours thérapeutique de la mère. Pour ce faire, les potentiels facteurs ont été sélectionnés au seuil de 20% en utilisant une régression logistique simple. Ils ont ensuite été introduits dans un modèle multivarié de régression logistique selon une stratégie pas à pas descendant afin d'obtenir des estimations ajustées. Le niveau de signification a été fixé à 5%. Les résultats ont été présentés sous forme d'Odds Ratio (OR) accompagnés de leurs intervalles de confiance à 95% (IC95%).

**Aspects éthiques:** les normes éthiques en matière de recherche, notamment l'anonymat et la confidentialité des données ont été respectées. Au préalable, les mères d'enfants retenus ont eu une note d'information détaillée sur l'étude. Pour les mères non instruites, nous avons procédé à une traduction de la note d'autorisation et du consentement éclairé en langues locales, sous une forme compréhensible pour expliquer le contexte et les raisons de l'étude. Les données d'enquête ont été collectées de façon individuelle après l'obtention du consentement oral, libre et éclairé de chaque cible enquêtée.

**Biais:** les agents de collecte et leurs superviseurs avaient reçu une formation d'une journée comprenant des modules sur plusieurs points: le processus de collecte des données, les techniques d'entretien, ainsi que les aspects éthiques. L'outil de collecte avait été préalablement testé dans 20 ménages non inclus dans l'étude. Les ajustements et les leçons tirées des questionnaires prétestés avaient servi à élaborer le questionnaire final. Une supervision rigoureuse, une vérification croisée quotidienne des questionnaires remplis avaient été réalisées pour garantir la qualité des données recueillies.

**Les limites:** l'étude étant transversale, on ne peut établir de relation causale entre les facteurs associés identités et le phénomène étudié (recours thérapeutique)

## Résultats

### Caractéristiques de base de la population d'étude

Au total, 448 mères d'enfants de moins de cinq ans ont été incluses dans l'étude. Elles étaient majoritairement non instruites/alphabétisées (47,10%) et de confession chrétienne (56,47%). Plus de neuf mères sur dix ont déclaré être en couple (96,88%) et avoir une activité génératrice de revenu (90,85%). Environ sept mères sur dix avaient connaissance d'un relais communautaire ; et moins du tiers avaient déjà entendu parler du PIHI Com. Environ 85% des mères disposaient de moustiquaire imprégnée d'insecticide (MII) dans leurs ménages. Par contre, près de la moitié des participantes à l'étude (47,77%) ne disposaient pas de latrines dans leurs domiciles. On notait que la majorité des mères (63,17%) vivaient dans des ménages de moins de six personnes. Parmi les mères enquêtées, 37,28% résidaient au sud du pays, 27,01% au centre et 35,71% au nord. Par ailleurs, pour plus de la moitié des mères, le plus jeune enfant était âgé de moins de 24 mois, de sexe masculin (51,12%) et de rang trois et plus (49,78%) (Tableau 1).

**Tableau 1:** caractéristiques sociodémographiques des mères d'enfants de moins de cinq ans, Bénin, 2022

Variables	n	%
**Niveau d'instruction de la mère**		
Non-instruite/alphabétisée	211	47,10
Primaire	111	24,78
Secondaire et plus	126	28,12
**Situation matrimoniale de la mère**		
Célibataire	14	3,13
Marié	434	96,87
**Activité génératrice de revenu de la mère**		
Non	41	9,15
Oui	407	90,85
**Religion de la mère**		
Aucune	13	2,9
Christianisme	253	56,47
Islam	112	25,00
Traditionnelle	70	15,63
**Connaissance de la mère d'un RC**		
Non	158	35,27
Oui	290	64,73
**Connaissance de la mère du PIHI Com**		
Non	311	69,42
Oui	137	30,58
**Taille du ménage**		
≤5	283	63,17
>5	165	36,83
**MII non-déchirés dans le ménage**		
Non	67	14,96
Oui	381	85,04
**Disponibilité de latrine/toilette dans le domicile**		
Non	214	47,77
Oui	234	52,23
**Région**		
Sud	167	37,28
Centre	121	27,01
Nord	160	35,71
**Age du plus jeune enfant (en mois):** 20 (10; 32)		
<12	125	27,90
12-23	125	27,90
24-35	89	19,87
36-47	65	14,51
48-59	44	9,82
**Sexe du plus jeune enfant**		
Féminin	219	48,88
Masculin	229	51,12
**Rang de naissance du plus jeune enfant**		
1	100	22,32
2	125	27,90
3 et plus	223	49,78

### Premier recours aux soins

**Figure 1:**
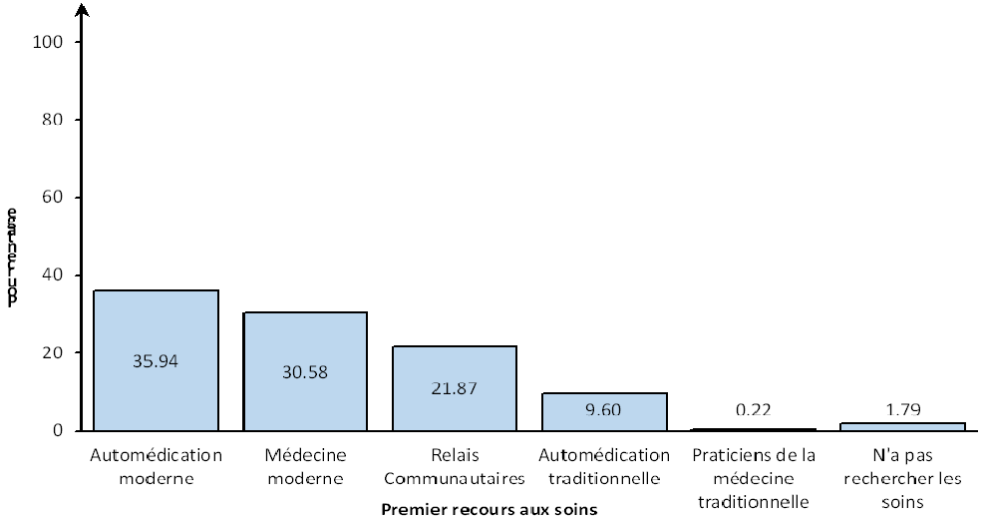
premier recours aux soins des mères d'enfants de moins de cinq ans, Bénin, 2022

Suite au dernier épisode morbide de leur plus jeune enfant, 52,46% des mères ont eu premièrement recours à la médecine moderne ou aux relais communautaires, contre 47,57% qui ont pratiqué une automédication (moderne ou traditionnelle), recouru aux praticiens de la médecine traditionnelle ou n'ont pas recherché les soins (Figure 1).

### Facteurs associés au premier recours aux soins

Le Tableau 2 met en évidence les facteurs associés au recours aux soins des mères d'enfants de moins de cinq ans à la médecine moderne ou aux relais communautaires en cas d'épisode morbide de leur plus jeune enfant. Les mères de confession chrétienne (ORa= 6,76 ; IC95%= 1,69-27,00) ou musulmane (ORa= 9,60; IC95%= 2,37-38,92) étaient plus susceptibles de faire recours à la médecine moderne ou à un relais communautaire par rapport à celles qui ont déclaré ne pas avoir de croyance religieuse. Les mères qui connaissaient des relais communautaires avaient 0,46 fois (IC95%= 0,29-0,73) de chance de les faire appel lorsque leurs enfants étaient malades ou de les envoyer dans une formation sanitaire en comparaison à celles qui ne les connaissaient pas. Les mères vivant dans des ménages ne disposant pas de latrine/toilette à domicile avaient 1,92 fois (IC95%= 1,19-3,08) plus de chance de faire appel à un relais communautaire ou de recourir à la médecine moderne pour un cas d'épisode morbide de leur enfant que celles qui en disposaient. En comparaison aux mères enquêtées au sud, celles habitant au nord (ORa=2 ; IC95%= 1,08 -3,68) étaient plus susceptibles de recourir à la médecine moderne ou aux relais communautaires (Tableau 2).

**Tableau 2:** facteurs associés au premier recours aux soins des mères d'enfants de moins de cinq ans, Bénin, 2022

Variables	Analyse univariée					Analyse multivariée				
	OR	IC95%			p	OR	IC95%			p
**Niveau d'instruction de la mère**										
Non-instruit/Alphabétisé	1,17	0,74	-	1,86	0,494					
Primaire	1,16	0,69	-	1,93	0,577					
Secondaire et plus	1									
**Situation matrimoniale de la mère**										
Célibataire	1									
Mariée	1,49	0,51	-	4,36	0,468					
**Activité génératrice de revenu de la mère**										
Non	0,76	0,40	-	1,45	0,412					
Oui	1									
**Réligion de la mère**										
Aucune	1					1				
Christianisme	3,36	0,90	-	12,50	0,071	6,76	1,69	-	27,00	0,007
Islam	8,33	2,15	-	32,27	0,002	9,60	2,37	-	38,92	0,002
Traditionnelle	1,85	0,47	-	7,36	0,381	2,89	0,69	-	12,18	0,149
**Connaissance de la mère d’un RC**										
Non	0,42	0,28	-	0,62	<0,001	0,46	0,29	-	0,73	0,001
Oui	1					1				
**Connaissance de la mère du PIHI Com**										
Non	0,52	0,35	-	0,79	0,002					
Oui	1									
**Taille du ménage**										
≤5	1									
>5	1,50	1,02	-	2,21	0,041					
**MII non-déchirés dans le ménage**										
Non	1,64	0,96	-	2,79	0,071					
Oui	1									
**Disponibilité de latrine dans le domicile**										
Non	2,88	1,96	-	4,23	<0,001	1,92	1,19	-	3,08	0,007
Oui	1					1				
**Age de l'enfant (en mois)**										
<12	1,12	0,56	-	2,22	0,749					
12-23	1,19	0,60	-	2,37	0,615					
24-35	1,34	0,65	-	2,77	0,426					
36-47	0,76	0,35	-	1,63	0,477					
48-59	1									
**Sexe de l'enfant**										
Féminin	1,08	0,74	-	1,56	0,688					
Masculin	1									
**Rang de naissance de l'enfant**										
1	1									
2	1,00	0,59		1,69	1,000					
5 et plus	1,43	0,89		2,30	0,136					
**Région**										
Sud	1					1				
Centre	1,08	0,67	-	1,73	0,750	1,16	0,69	-	1,95	0,571
Nord	3,23	2,05	-	5,11	<0,001	2,00	1,08	-	3,68	0,027

## Discussion

Les résultats de ce travail mettent en évidence les facteurs associés au premier recours aux soins des mères d'enfants de moins de cinq ans. Le premier recours thérapeutique des mères était différent selon la région de résidence. Cependant, malgré les inégalités régionales exposées, un meilleur accès aux services médicaux peut favoriser une moindre automédication [13]. En effet, la comparaison des mères enquêtées au sud, par rapport à celles enquêtées au nord met en évidence que les mères d'enfants vivant au nord étaient plus susceptibles de faire recours à la médecine moderne ou aux relais communautaires. Ce résultat pourrait être expliqué par la densité populationnelle qui entraîne les longues files d'attente, facteurs de réticence des femmes aux services de santé au sud [14]. Aussi, la cherté de la vie et l'accès facile de la population du sud aux médicaments à travers la disponibilité des pharmacies dans la quasi-totalité des villes ou à la proximité des domiciles pourraient être à l'origine du non-recours à la médecine moderne [15].

Dans notre étude, la religion a été identifiée comme un facteur associé au premier recours aux soins au Bénin. En effet, les mères de confession chrétienne ou musulmane étaient plus susceptibles de faire recours à la médecine moderne ou à un relais communautaire par rapport à celles qui ont déclaré ne pas avoir de croyance religieuse. Ce résultat est contraire à celui trouvé par Kifle *et al*. [16]. Dans leur étude, les musulmans, les protestants étaient plus susceptibles de pratiquer l'automédication [16]. Ces disparités observées dans les études peuvent s'expliquer par le fait que les cibles ne soient pas de la même catégorie. L'étude de Kifle *et al*. portait sur les infirmiers et les étudiants, en occurrence, les étudiants en pharmacie, ce qui n'est pas le cas dans notre travail. Avoir connaissance d'un relais communautaire est une chance pour recourir aux services de santé en cas de problème de santé. Les mères qui connaissaient les relais communautaires avaient 0,46 fois plus de chance de leurs faire appel lorsque leurs enfants étaient malades ou de les envoyer dans une formation sanitaire en comparaison à celles qui ne les connaissaient pas. Ce résultat rejoint celui décrit par Renaud qui affirme que la communication pour la santé a pour but non seulement d'informer, mais de sensibiliser les individus, les organisations et les communautés aux questions relatives à la santé. La communication et l'éducation sur les prestations et services de santé sont des facteurs clés de la réussite dans l'utilisation des services de santé de base. Il en découle l'amélioration de l'engagement communautaire [17].

Les mères vivant dans des ménages ne disposant pas de latrine/toilette à domicile avaient 1,92 fois plus de chances de faire appel à un relais communautaire ou de recourir à la médecine moderne par rapport à celles qui en disposaient. Le comportement des mères ne disposant pas de latrines s'expliquerait par le fait que ces ménages sont confrontés dans la plupart des cas, aux épisodes des infections récurrentes et des maladies diarrhéiques [18,19]. Le niveau de la gravité des cas et des signes cliniques effrayant tels que la diarrhée associée au vomissement pourrait contraindre les mères à recourir le plus rapidement possible à la médecine moderne au risque de perdre leur enfant ; ce qui leur donne probablement plus l'habitude de fréquenter les relais communautaires ou la médecine moderne. Les manipulateurs d'aliments et les femmes de chambre infectés asymptomatiques sont une source potentielle d'infection pour la communauté avec de nombreux parasites intestinaux et d'autres agents infectieux entéropathogènes très souvent retrouvés dans des toilettes non hygiéniques [20].

Dans notre étude l'ordre du premier recours est l'automédication moderne, la médecine moderne, le RC, l'automédication traditionnelle, n'a pas recherché de soins et le recours aux praticiens de la médecine traditionnelle tandis que Houéto *et al*. ont trouvé que l'ordre de recours est le domicile, les voisins, le guérisseur et les centres de santé dans leur étude [21]. Il faut noter que le premier recours n'est pas le système de santé comme le recommande la conférence d'Alma Ata pour assurer aux populations l'accès aux soins de santé primaires [22].

La réduction des facteurs qui constituent un goulot d'étranglement au recours au système de santé en premier intention des mères ou gardiennes d'enfants de moins de 5ans se présente comme une solution incontournable aux défis liés à l'accès de la population à la prise en charge rapide. Les stratégies de communication sur les comportements préventifs en matière de promotion de la santé, la réduction des inégalités sociales et la bonne gestion des dépenses de santé représentent les pistes de solution au recours systématique au système de santé en cas de maladie. Les facteurs identifiés soulignent l'importance d'intervenir sur des éléments modifiables tels que la promotion de l'action des relais communautaires pour améliorer le recours à leurs prestations.

## Conclusion

Il est apparu que près de la moitié des mères préfèrent opter premièrement pour une automédication (moderne ou traditionnelle), aux praticiens de la médecine traditionnelle ou ne recherchent pas les soins en cas d'épisode morbide de leur plus jeune enfant. Ces résultats soutiennent la nécessité d'agir sur les facteurs modifiables tels que la connaissance du relais communautaire par sa communauté afin de susciter la participation et l'engagement communautaire. L'organisation des séances d'apprentissage et d'échange permettra de partager plusieurs informations sur le système de santé et la mise en œuvre de ses activités. Il s'agira de contextualiser les séances selon les régions et les cultures au lieu de les réaliser de façon traditionnelle (plaidoyers, causeries éducatives, mobilisation sociale…). Aussi, l'utilisation des canaux de communications telles que les radios, les télévisions, les réseaux sociaux pour informer la communauté de l'existence des RC ou ASC et les différentes prestations qu'ils peuvent offrir permettra de renforcer leur implication dans la communauté et réduire les inégalités sociales relatives à l'accès aux prestations de services et aux soins des communautés à la base.

### Etat des connaissances sur le sujet

- Le besoin de montrer aux mères d'enfant des 0 à 5 ans l'importance du recours au système de santé;
- Le comportement des mères d'enfants de 0 à 5 ans par rapport au recours aux soins imputables à plusieurs évènements de santé.

### Contribution de notre étude à la connaissance

- L'étude met en exergue le comportement (le premier recours) des mères d'enfants de 0 à 5 ans face à un danger (l'épisode morbide de leur enfant);
- Les facteurs associés qui pourraient expliquer leur première réaction pour une prise de décision au niveau stratégique qui facilite la disponibilité des soins à proximité c'est-à-dire en communauté à travers le travail des agents de santé communautaires (sensibilisation à la prévention, visite à domicile, causerie éducative contextualiser selon chaque région, des relais communautaires…).

## Remerciements

Nous remercions toutes les personnes qui ont accepté de participer à cette étude, ainsi que les enquêteurs sans qui celle-ci n'aurait pas été possible.
